# Spatiotemporal Variations of Particulate and Gaseous Pollutants and Their Relations to Meteorological Parameters: The Case of Xiangyang, China

**DOI:** 10.3390/ijerph17010136

**Published:** 2019-12-24

**Authors:** Wei Xue, Qingming Zhan, Qi Zhang, Zhonghua Wu

**Affiliations:** 1 School of Urban Design, Wuhan University, Wuhan 430072, China; 2 Bank of Communications, Wuhan 430015, China; 3 The Xiangyang Environmental Monitoring Center, Xiangyang 441000, China

**Keywords:** air pollutants, spatiotemporal variations, holiday effect, meteorological parameters, a prefecture-level city

## Abstract

High air pollution levels have become a nationwide problem in China, but limited attention has been paid to prefecture-level cities. Furthermore, different time resolutions between air pollutant level data and meteorological parameters used in many previous studies can lead to biased results. Supported by synchronous measurements of air pollutants and meteorological parameters, including PM_2.5_, PM_10_, total suspended particles (TSP), CO, NO_2_, O_3_, SO_2_, temperature, relative humidity, wind speed and direction, at 16 urban sites in Xiangyang, China, from 1 March 2018 to 28 February 2019, this paper: (1) analyzes the overall air quality using an air quality index (AQI); (2) captures spatial dynamics of air pollutants with pollution point source data; (3) characterizes pollution variations at seasonal, day-of-week and diurnal timescales; (4) detects weekend effects and holiday (Chinese New Year and National Day holidays) effects from a statistical point of view; (5) establishes relationships between air pollutants and meteorological parameters. The principal results are as follows: (1) PM_2.5_ and PM_10_ act as primary pollutants all year round and O_3_ loses its primary pollutant position after November; (2) automobile manufacture contributes to more particulate pollutants while chemical plants produce more gaseous pollutants. TSP concentration is related to on-going construction and road sprinkler operations help alleviate it; (3) an unclear weekend effect for all air pollutants is confirmed; (4) celebration activities for the Chinese New Year bring distinctly increased concentrations of SO_2_ and thereby enhance secondary particulate pollutants; (5) relative humidity and wind speed, respectively, have strong negative correlations with coarse particles and fine particles. Temperature positively correlates with O_3_.

## 1. Introduction

Air pollution is caused by manifold particulate and gaseous pollutants, namely, particles with aerodynamic diameter less than 2.5 μm (PM_2.5_) and less than 10 μm (PM_10_), and total suspended particles (TSP), carbon monoxide (CO), nitrogen dioxide (NO_2_), ozone (O_3_) and sulfur dioxide (SO_2_) [1]. Prolonged high levels of air pollution have been verified to have significant adverse impacts on human health: (1) particulate matters, especially fine particles, can increase the risk of cerebrovascular diseases, heart diseases, chronic obstructive pulmonary diseases (COPD), acute respiratory infections and lung cancer [2,3,4,5]; (2) intensive exposures to O_3_ and NO_2_ can lead to COPD, respiratory morbidity and premature death [2,5,6,7]; (3) high levels of CO and SO_2_ can cause cataracts, respiratory symptoms and COPD [2,8,9]. According to the World Health Statistics 2018 released by the World Health Organization, 7 million people are killed annually by air pollution-related diseases all over the world, and in China the number is around 1.83 million [3]. Substantial efforts to clarify air pollution distributions, and thereby to alleviate haze problems desperately need to be made.

A ground monitoring network was established by the Ministry of Ecology and Environment of China (MEEC) on 2000, and then was expanded to monitor 338 cities, with measurements of PM_2.5_, PM_10_, CO, NO_2_, O_3_ and SO_2_, on 2015 [10,11]. Subsequently, remarkable progress has been achieved on spatiotemporal variations of air pollutants at urban scale. Spatially, previous studies primarily focused on a specific city or comparisons between cities. Due to unevenly distributed ground monitoring stations, most investigations were carried out in key cities or environment protection mode cities [12,13,14], albeit with limited studies on fine particulate matters and air quality index (AQI) in prefecture-level cities [15,16]. Equivalent findings in temporal variabilities of air pollutants have shown interannual, seasonal, day-of-week, diurnal and specific timescale variability characteristics (i.e., haze episodes and national holidays) [15,16,17,18,19,20,21,22,23]. Nevertheless, TSP has not popularly been studied since 2001 when TSP was excluded from the China Environment Bulletin [23]. A distinct seasonal pattern for “worse winter and better summer” and traffic-related diurnal characteristics were captured by an array of studies [15,16,17,18,19,20,21,22]. Although day-of-week variations in different cities follow different rules, mean concentrations of air pollutants during weekdays are usually greater than those during weekends. However, seldom studies confirmed the weekday-weekend difference of pollution concentrations from a statistical point of view. In terms of discovering variations of pollutant concentrations during national holidays, such as the Chinese New Year (CNY), some studies have revealed a clear holiday effect which is defined as differences of air pollution concentrations during national holidays and non-holiday periods by using the independent samples t-test and paired samples t-test [24]. However, the non-CNY period, covering 10 days before and 10 days after CNY, is not applicable to mainland China, because a compact but prodigious population mobility that can be observed during that period, suggesting more traffic-related emissions than usual, which in turn can disrupt the holiday effect test results.

Regarding the meteorological influences, some valuable results were unveiled thanks to the open data collected by the China Meteorological Administration (CMA). Specifically, certain synoptic conditions, such as low wind speed (WS) and stagnant weather, can increase the risk of haze occurrence while strong windy and rainy weathers reasonably disrupt accumulation as well as scavenge of particles [18,19,21,23]. Meanwhile, temperature, sunshine duration and planetary boundary layer height (PBLH) are generally believed to accelerate the local photochemical formation, and thereby bring heavy secondary generated O_3_ pollution [18,19,21,23]. However, the publicly accessible measurements for meteorological parameters (i.e., pressure, temperature, relative humidity, precipitation, evaporation, wind speed and direction, sunshine duration, and ground surface temperature) from the CMA, on a daily time scale, are insufficient to synchronously detect meteorological effects due to the hourly variations of air pollutants. The problem of different time resolutions has been noted by some scholars [18,25]. Furthermore, air pollutants and meteorological parameters for the same city are normally measured by different stations situated at different locations, which probably generates biased results for the meteorological influences on air pollution distributions.

Supported by synchronous ground measurements of air pollutants and meteorological parameters (i.e., PM_2.5_, PM_10_, TSP, CO, NO_2_, O_3_, SO_2_, temperature, relative humidity, wind speed and direction), at 16 urban sites in Xiangyang from 1 March 2018 to 28 February 2019, we first analyzed the overall air quality with AQI and then captured the spatial dynamics of air pollutants with ten-kind point pollution source data. Thirdly, we characterized pollution variations on a seasonal, day-of-week and diurnal time scale, including the diagnoses of weekend effects and holiday effects by the Kruskal-Wallis rank-sum test. Finally, the Spearman’s rank correlation coefficient was strictly applied to establish relationships between air pollutants and meteorological parameters.

## 2. Materials and Methods

### 2.1. Study Area and Measurement Stations

**Figure 1:**
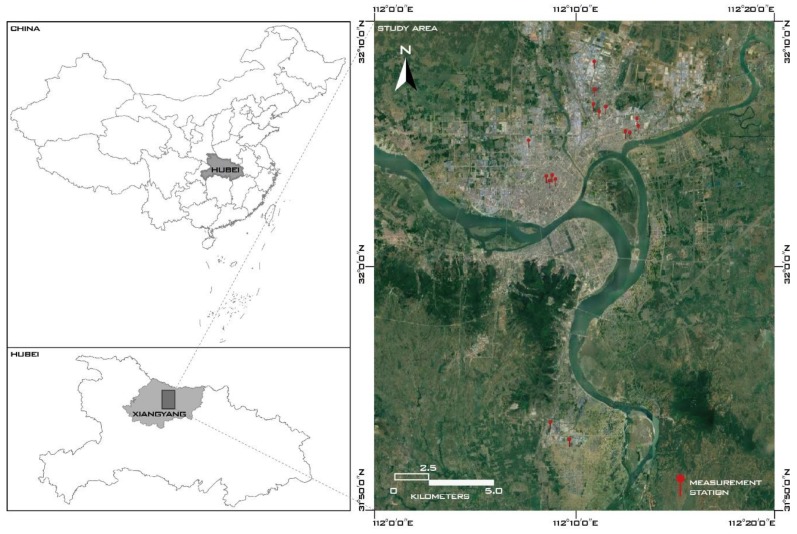
The locations of the study area and 16 measurement stations.

Xiangyang is a prefecture-level city lying on the middle reaches of the Han River in central China (Figure 1), and features a subtropical monsoon climate all the year round. According to the Xiangyang Statistical Yearbook 2018, half of the Gross Domestic Product (GDP) of Xiangyang is derived from industrial production, especially from automobile manufacturers and chemical industries [26]. Consequently, severe haze episodes have been observed more frequently here. To clarify the agglomeration and diffusion mechanism of air pollution, an hourly monitoring network of air pollutants and meteorological parameters was established in the built environment of Xiangyang by the Xiangyang Environmental Monitoring Center in February 2018.

The hourly monitoring system includes 16 measurement stations which automatically record data of seven air pollutants and four meteorological parameters, namely, particles with aerodynamic diameter less than 2.5 μm (PM_2.5_) and less than 10 μm (PM_10_), total suspended particles (TSP), carbon monoxide (CO), nitrogen dioxide (NO_2_), ozone (O_3_), sulfur dioxide (SO_2_), temperature, relative humidity (RH), wind speed (WS) and wind direction (WD). Based on the Chinese Ambient Air Quality Standard (GB3095-2012) and the Specifications for Surface Meteorological Observation (GB/T 35226-2017 and GB/T 35227-2017), the sampling methods are as follows: (1) tapered element oscillating microbalances together with the *β*-ray method are adopted to measure the mass concentrations of PM_2.5_, PM_10_ and TSP; (2) the non-dispersion infrared absorption method together with the gas filter correlation infrared method is utilized to measure the mass concentration of CO; (3) the differential optical absorption spectroscopy (DOAS) method together with the chemiluminescence method is employed to measure the mass concentration of NO_2_; (4) the DOAS method together with the ultraviolet fluorescence method is used to measure the mass concentrations of O_3_ and SO_2_; (5) AQMS2000 monitors (the Fairsense Ltd., Beijing, China) are installed to observe temperature, RH, WS and WD [27,28,29]. Installation and acceptance of measurement stations rely on the technical guidelines of GB/T 35226-2017, GB/T 35227-2017, HJ655-2013 and HJ193-2013 [28,29,30,31].

### 2.2. Data Sources

#### 2.2.1. Data of Air Pollutants and Meteorological Parameters

In this study, we adopted continuous twelve-month data of seven air pollutants and four meteorological parameters collected by the 16 aforementioned measurement stations from 1 March 2018 to 28 February 2019. To maintain data validity in the subsequent analyses, a data check in compliance with the Chinese Ambient Air Quality Standard [27] was conducted on the platform of R, and we found some missing values for TSP, WS and WD of Station 15 and Station 16, further demonstrated to be the storage loss. Statistical summaries of hourly measurements for four meteorological parameters and seven air pollutants during the studied period were respectively listed in Table 1 and Table 2.

Notably, the data of daily urban wind speed from 1 March 2018 to 28 February 2019 downloaded from the website of CMA [32] was used to make a comparison with the WS measured by those 16 measurement stations. In addition, Station 15 and Station 16 were excluded from the subsequent analyses related to TSP variations as well as correlations between air pollutants and meteorological parameters.

As a non-dimensional index, the AQI is broadly utilized to reveal levels of air pollution and risks to human health [33,34]. In this study, the category and the calculation of AQI are based on technical specifications described by the MEEC in the HJ633-2012 (Table 3) [35]. Generally, the higher the AQI number, the worse the air condition. Equations for AQI are as follows:

(1) $$IAQI_{p}=\frac{IAQI_{Hi}-IAQI_{Lo}}{BP_{Hi}-BP_{Lo}}\left(C_{p}-BP_{Lo}\right)+IAQI_{Lo},$$

(2) $$AQI=max\left\{IAQI_{1},IAQI_{2},IAQI_{3},\ldots ,IAQI_{6}\right\}$$

where $IAQI_{p}$ represents the individual air quality index for pollutant p; $C_{p}$ is the mass concentration for pollutant p; $BP_{Hi}$ and $BP_{Lo}$ respectively denote the closest upper and lower breakpoint of $C_{p}$; $IAQI_{Hi}$ and $IAQI_{Lo}$ respectively represent the IAQI corresponding to $BP_{Hi}$ and $BP_{Lo}$. Detailed technical specifications of thresholds for each pollutant can be found in the HJ633-2012 [35]. Equation (2) illustrates the overall AQI number determined by the maximum of 6 IAQIs including IAQI for PM_2.5_, PM_10_, TSP, CO, NO_2_, O_3_ and SO_2_. Meanwhile, the individual pollutant with the maximal value is defined as the primary pollutant of that day when the daily AQI number is above 50. Finally, we put the mass concentration for each pollutant in Equations (1) and (2) to obtain the daily AQI and the primary pollutant from 1 March 2018 to 28 February 2019.

#### 2.2.2. Pollution Point-Source Data 

Stationary pollution sources are acknowledged to have certain impacts on the spatiotemporal distribution of air pollutants. Therefore, 10 kinds of point source data, including feed mills, automobile manufacturers, automobile repair workshops, machinery manufacturers, chemical plants, electronic companies, material companies, building material companies, other industrial companies, and on-going construction sites were used to depict the surrounding built environment of each measurement station.

Locations of the industrial facilities were obtained from the Xiangyang Natural Resources and Planning Bureau (XNRPB) [36] and Google Maps (the Google LLC, Mountain View, CA, US). The list of on-going construction sites was collected from the website of Xiangyang Housing and Urban-Rural Development Bureau [37]. Then, we pinpointed them on the Google Map. Moreover, 16 buffer zones of measurement stations were created, and the optimal radius of the buffer zone was demonstrated to be 1 km by prior studies [38,39].

### 2.3. Methods

Strong winds or rainfalls can have scavenging effects on atmospheric pollutants, so days with daily WS over 3.4 m/s or daily precipitations over 5 mm were excluded from the subsequent analyses [40]. In addition, holidays can also disrupt the results. Specifically, most work plants do not produce on weekends, and if a Saturday or a Sunday happens to be a working day, the final mean concentration of air pollutants on Saturday or Sunday will probably be higher than the true value. Thus, if a day from Monday to Friday happens to be a holiday or if a Saturday or a Sunday happens to be a working day, we excluded that day from the analyses for the day-of-week variations. Other notable details in this study were listed here: (1) particulate pollutants encompass PM_2.5_, PM_10_ as well as TSP, and air pollutants stand for particulate pollutants along with gaseous pollutants; (2) the data of WS, temperature and RH participating in the Spearman’s rank test were collected by the 16 measurement stations.

Daily mean concentrations were averaged by hourly measurements, and monthly mean concentrations were respectively averaged by daily and monthly mean concentrations. Analogously, we obtained the mean concentrations of air pollutants for diurnal analysis. Spatially, the annual mean concentrations of air pollutants for each measurement station were averaged by using the corresponding daily mean concentrations. CNY and National Day (NDH) holidays are the equally longest holidays (7 days) in mainland China, and significant behavioral changes from usual days can be observed during CNY and NDH. Specifically, CNY is the most important holiday for Chinese people to get together, resulting in a compact but prodigious population mobilization from a week before to a week after CNY. Meanwhile, many work plants and construction sites are closed for several days during CNY and NDH. Moreover, celebration activities (i.e., setting off firecrackers, burning paper money to worship ancestors on New Year’s Eve, etc.), temporarily permitted by the government, can also be observed during the CNY. To sum it up, with reduced local stationary emissions, CNY and NDH are suitable study periods to conduct a diagnosis of the holiday effect [24,41]. To rule out meteorological influences, we defined the non-holiday period for CNY (non-CNY period) as the time interval from 1 December 2018 to 28 February 2019 excluding CNY and the non-holiday period for NDH (non-NDH period) as the time interval from 1 September 2018 to 30 November 2018 excluding NDH. Then, we discovered that not all data of air pollutants comply with the normal distribution by carrying out the Kolmogorov-Smirnov test. Given the fact that the Kruskal-Wallis rank-sum test is a widely used nonparametric approach to examine differences between multi-independent group samples with unknown distributions [16], it was introduced in our study after a Chi-square test confirming the seven air pollutants are independent from each other. Subsequently, we conducted the Kruskal-Wallis test at the significant level of 5% and the null hypothesis ($H_{0}$) is no significant differences of distributions of air pollutants during holidays and non-holiday period. When the p value of this test is less than 0.05, the $H_{0}$ will be rejected, suggesting an obvious holiday effect, and vice versa. We repeated the same approach to examine the weekday-weekend statistical difference of air pollution concentrations. Finally, the Spearman’s rank correlation coefficient was introduced to identify relationships between air pollutants and meteorological parameters. Notably, the Kruskal-Wallis test was realized on the platform of R and the Kolmogorov-Smirnov test as well as the Spearman’s rank test was processed on the MATLAB R2015b (the MathWorks Inc., Natick, MA, US).

## 3. Results and Discussion

### 3.1. Overview of Air Quality

**Figure 2:**
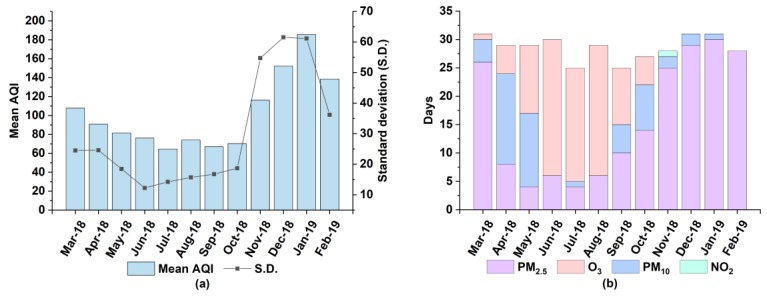
Characteristics of the air quality index (AQI) and the primary pollutants to AQI in Xiangyang by months from March 1, 2018 to February 28, 2019: (**a**) AQI; (**b**) primary pollutants.

The annual AQI average in Xiangyang on all measurements from 1 March 2018 to 28 February 2019 is 102.0 ± 51.2. The monthly AQI average exhibited a cosinoidal pattern, as shown in Figure 2. The air quality keeps ameliorating after March, and then maintains a steadily good condition from July to October. Nevertheless, the air quality deteriorates after October, and reached the worst condition in January, 2019. Regarding the percentage of the days with different air quality, the result was ranked in a decreasing order: excellent and good air quality day (64.6%) > slightly polluted day (21.5%) > moderately polluted day (7.9%) > heavily polluted day (6.0%) > severely polluted day (0). The percentage of days with excellent and good air quality fails in reaching the criteria of 80% proposed by China’s 13th Five-Year Plan (2016–2020). Furthermore, in order to better understand the contribution of air pollutants to the AQI, the primary pollutants over the same time interval were calculated by months (Figure 2). The year-round air quality in Xiangyang mainly deteriorates due to the joint effect of three primary pollutants, namely, PM_2.5_, O_3_ and PM_10_, accounting for 52.1%, 27.4% and 14.2% respectively. PM_2.5_ and PM_10_ act as the primary pollutant all the year round, by contrast, O_3_ loses its position of primary pollutant since November. The main reason for O_3_ to be considered as the dominant primary pollutant in summer is that high temperature, along with more hours of sunlight, helps accelerate the local photochemical formation [11,14,42]. The contingently one-time occurrence of NO_2_ as the primary pollutant might be the result of a long-range transported pollution from neighboring regions. Compared with prior studies in Beijing, SO_2_ is not one of primary pollutants even in winter. The variability is probably derived from coal-fired heating extensively used during winter in the north of China [1].

### 3.2. Spatial Variations of Particulate and Gaseous Pollutants

**Figure 3:**
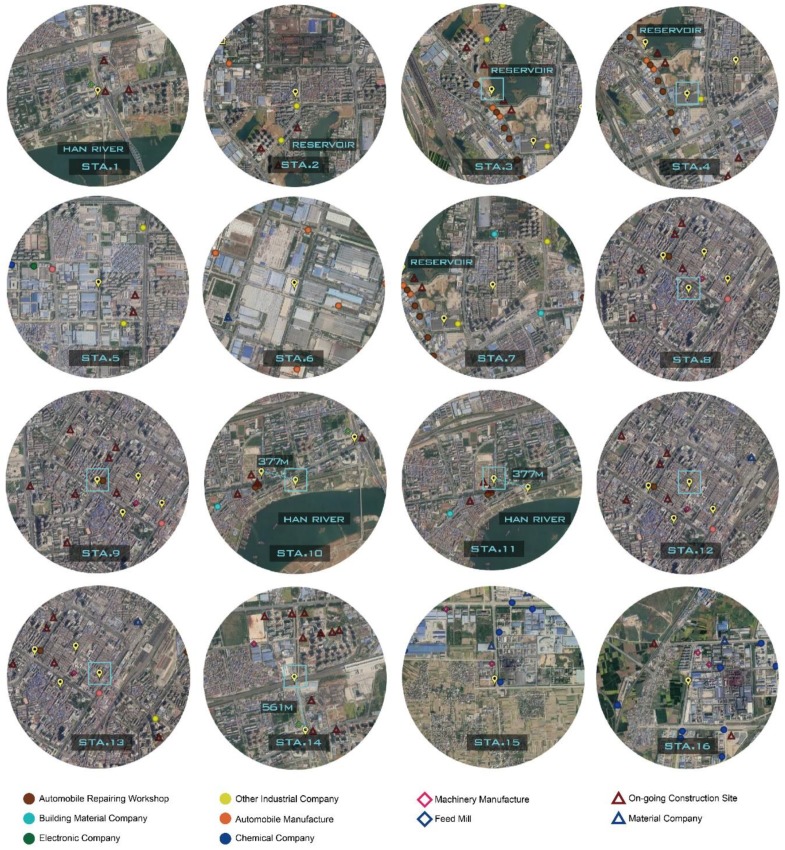
Pollution sources within one-kilometer-radius buffer zones of 16 measurement stations.

The locations of 16 measurement stations and their annual concentrations of air pollutants are displayed in Figure 3 and Table 4. Obviously, annual concentrations of PM_2.5_ and PM_10_ measured by all stations strikingly exceed both the Chinese National Ambient Air Quality Standards (CNAAQS) [27] and the World Health Organization Air Quality Guidelines (WHOAQG) [43]. Spatially, the largest annual values of gaseous pollutants are identically reported by Station 15 while the maximal annual concentrations of particulate pollutants are from Station 6. The highest level of each gaseous pollutant of Station 15 is caused by nine nearby chemical plants. However, Station 16, also surrounded by nine chemical plants, has relatively less concentrations of gaseous pollutants when compared to Station 15. By field investigation, we figured out that the difference is mainly ascribed to the location of the monitor. The monitor of Station 15 is inside a fertilizer factory, whereas the monitor of Station 16 is 10 m away from the factory gate.

The most abundant concentrations of particulate pollutants of Station 6 are closely associated with the land use made by automobile manufacture, accounting for four automobile work plants, three machinery factories and two electronics factories. It also suggests that automotive plants contribute to more particulate pollutants rather than gaseous pollutants while chemical plants emit more gaseous pollution. Meanwhile, Station 2 recorded the minimal annual concentrations of particulate pollutants and SO_2_ due to its location in a residential area. In addition, interesting results of heterogeneous variations for air pollutants between some adjacent stations were uncovered. Specifically, Station 3 and Station 7 (distance of 500 m) as well as Station 14 and Station 1 (distance of 565 m) show relatively uniform annual variations of gaseous pollutants while vary from annual concentrations of particulate pollutants (especially TSP). Different numbers of on-going construction sites are responsible for the variability. Specifically, Station 3 and Station 14 with more on-going construction sites inevitably produce more resuspended dust. It also can be seen that Station 10 and Station 11 (distance of 377 m) have the same number of on-going construction sites, but Station 11 monitors larger TSP concentrations, as Station 11 closer to construction sites. Station 9, Station 8, Station 11 and Station 13 also have several on-going construction sites in the buffer zone, but their annual concentrations of TSP are relatively lower. With reference to the urban management information on the website of XNRPB [36], one possible reason is that road sprinkler operations help deposit TSP.

### 3.3. Temporal Variations of Particulate and Gaseous Pollutants

#### 3.3.1. Seasonal Characteristics

**Figure 4:**
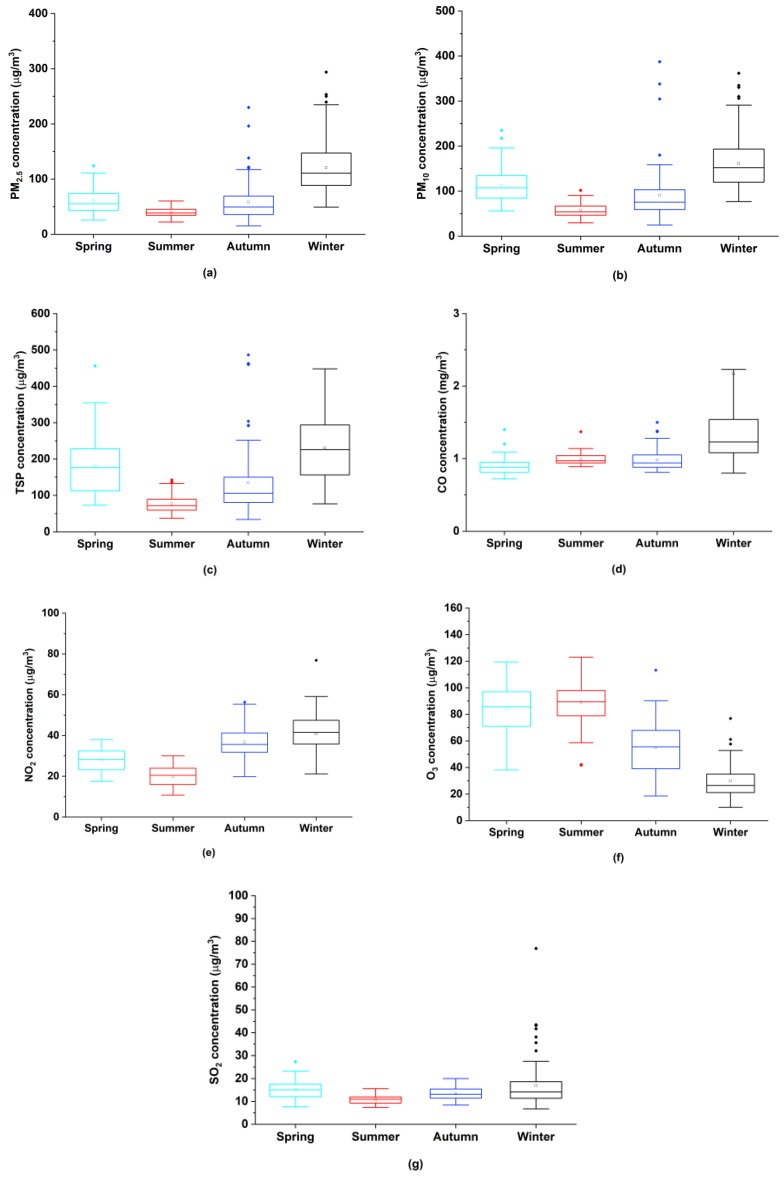
Seasonal variations of air pollutants in Xiangyang from March 1, 2018 to February 28, 2019: (**a**) PM_2.5_; (**b**) PM_10_; (**c**) total suspended particles (TSP); (**d**) CO; (**e**) NO_2_; (**f**) O_3_; (**g**) SO_2_.

Seasonal averages of PM_2.5_, PM_10_, TSP, NO_2_ and SO_2_ similarly exhibit U-shaped patterns with the common feature of the maximum being in winter while the minimum being in summer. Conversely, the seasonal mean concentration of O_3_ interestingly presents an inverted U-shaped pattern (Figure 4). The reason for the phenomenon is that lower temperature and less hours of sunlight in winter are unfavorable to photochemical activities, and then interfere with the O_3_ formation. Meanwhile, lower PBLH along with stagnant synoptic conditions in winter has adverse effects on particles diffusion [1,14,44]. The seasonal average of O_3_ slightly increases from spring to summer, and then strikingly decreases from summer to winter. This echoes the fact that O_3_ loses its position of primary pollutant since November as previously discovered. By contrast, the seasonal mean concentration of CO maintains a relatively stable level and a slight peak appears in winter.

#### 3.3.2. Concentration Changes during Chinese New Year and National Day Holidays

**Figure 5:**
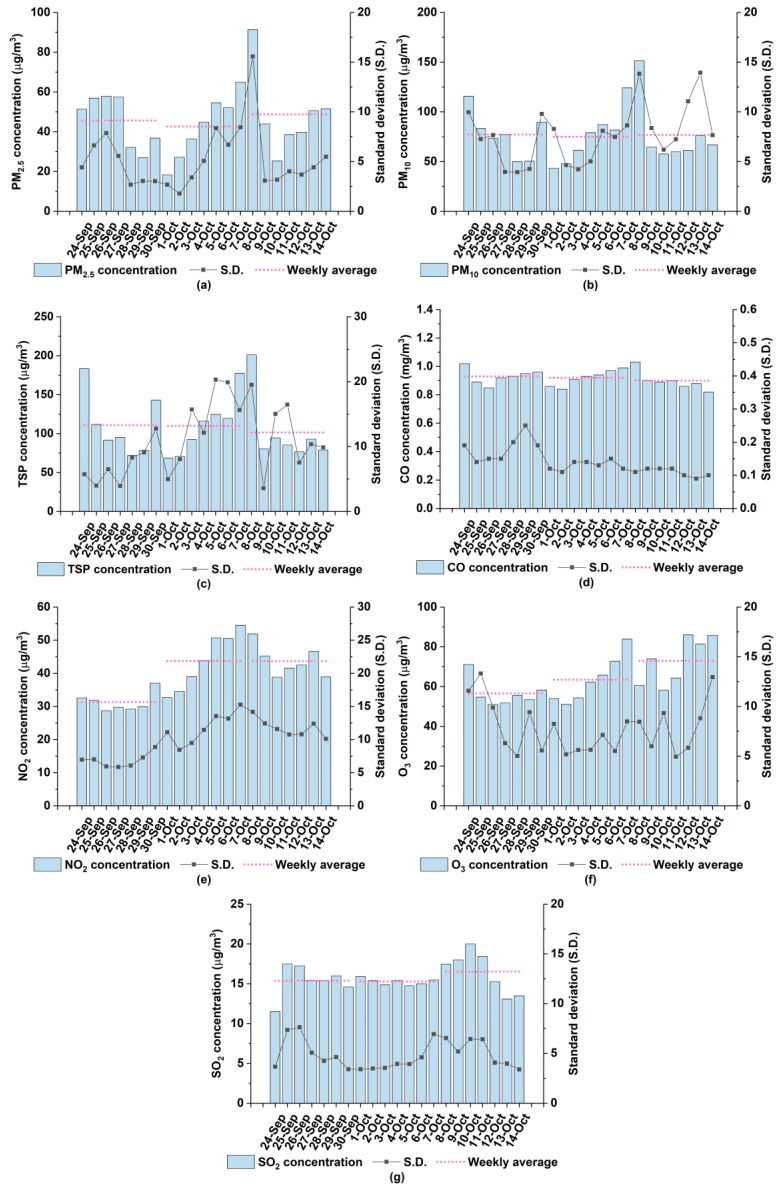
Daily variations of mean concentrations for air pollutants in Xiangyang from a week before National Day holidays to a week after National Day holidays: (**a**) PM_2.5_; (**b**) PM_10_; (**c**) TSP; (**d**) CO; (**e**) NO_2_; (**f**) O_3_; (**g**) SO_2_.

The results of the difference tests of NDH-nonNDH and CNY-nonCNY on all measurements were listed in Table 5. Significant differences of concentrations of NO_2_ and SO_2_ during NDH and non-NDH were uncovered. Specifically, the mean concentrations of NO_2_ and SO_2_ during NDH are higher than those during non-NDH, despite many plants being off-line during NDH. To further illustrate the results, we visualized the daily and weekly variations from a week before NDH to a week after NDH (24 September 2018 to 14 October 2018) in Figure 5. Generally, during NDH, the mean concentrations of PM_2.5_, PM_10_, TSP, NO_2_ and O_3_ respectively show a similarly dramatic uptrend and the mean concentration of CO exhibits a slight increase. However, the mean concentration of SO_2_ interestingly maintains a stable level. Notably, concentrations of all air pollutants reach the peak during the period from October 7 to October 9. After having checked the wind direction and air pollution information of neighboring provinces, we discovered that the higher concentrations during NDH can probably be ascribed to the pollution transported by the northeast wind from Henan Province.

**Figure 6:**
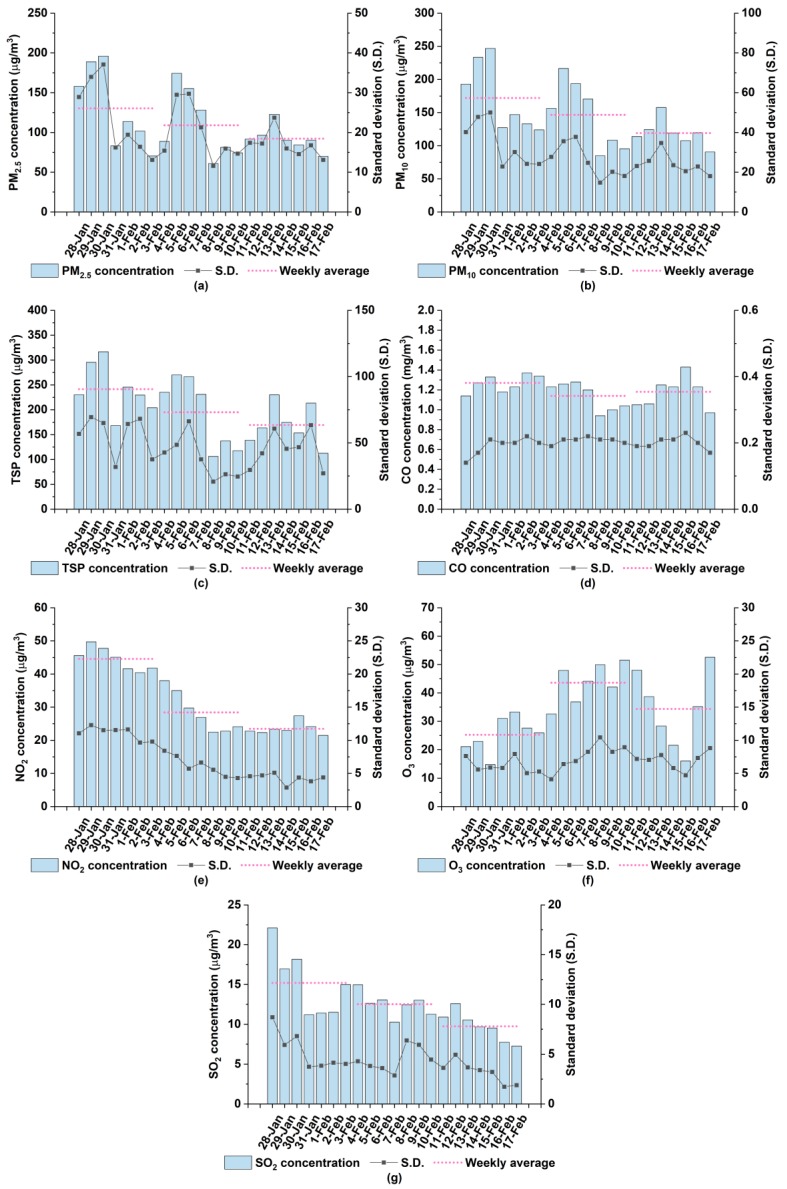
Daily variations of mean concentrations for air pollutants in Xiangyang from a week before Chinese New Year to a week after Chinese New Year: (**a**) PM_2.5_; (**b**) PM_10_; (**c**) TSP; (**d**) CO; (**e**) NO_2_; (**f**) O_3_; (**g**) SO_2_.

Significant differences of the NO_2_ concentration and the O_3_ concentration during CNY and non-CNY were unveiled, and the mean concentration of NO_2_ during non-CNY is much greater than that during CNY. The result is primarily ascribed to suspended industrial productions, engineering constructions, etc. during CNY, suggesting reduced stationary emissions over that time interval. It also can be seen from Figure 6 that trends of mean concentrations for PM_2.5_, PM_10_, TSP, CO and SO_2_ during CNY fluctuate like a sinusoid, and drastically climb to the maximum on February 4 or February 5. Specifically, the mean concentration of SO_2_ peaks on February 4 and particulate pollutants peak on February 5. Given the fact that 4 February 2019 is the eve of CNY, certain celebration activities, such as setting off firecrackers to welcome the new year and burning paper money to honor ancestors, happen on that day, which magnify SO_2_ concentration and subsequently cause an abundance of secondary particulate pollution.

#### 3.3.3. Day-of-Week Characteristics

Large cohort studies have investigated on the day-of-week variation by averaging every Monday to Sunday concentration for each air pollutant during the studied period, and they found that the mean concentration of a weekday (such as Wednesday) was higher than that of a weekend day (Saturday or Sunday) [15,17,19,20]. In our study, to clarify whether pollution concentrations are significantly heavier during weekdays than those during weekends, we performed the Kruskal-Wallis test at the significant level of 5% and the null hypothesis ($H_{0}$) is no significant differences of distributions of air pollutants during holidays and non-holiday period. It can be seen from the Table 6 that all *p* values of the difference test are larger than 0.05, suggesting no significant weekday-weekend statistical difference in Xiangyang during the studied period. That is to say, unclear weekend effect for all air pollutants during the studied period is confirmed.

#### 3.3.4. Diurnal Characteristics

**Figure 7:**
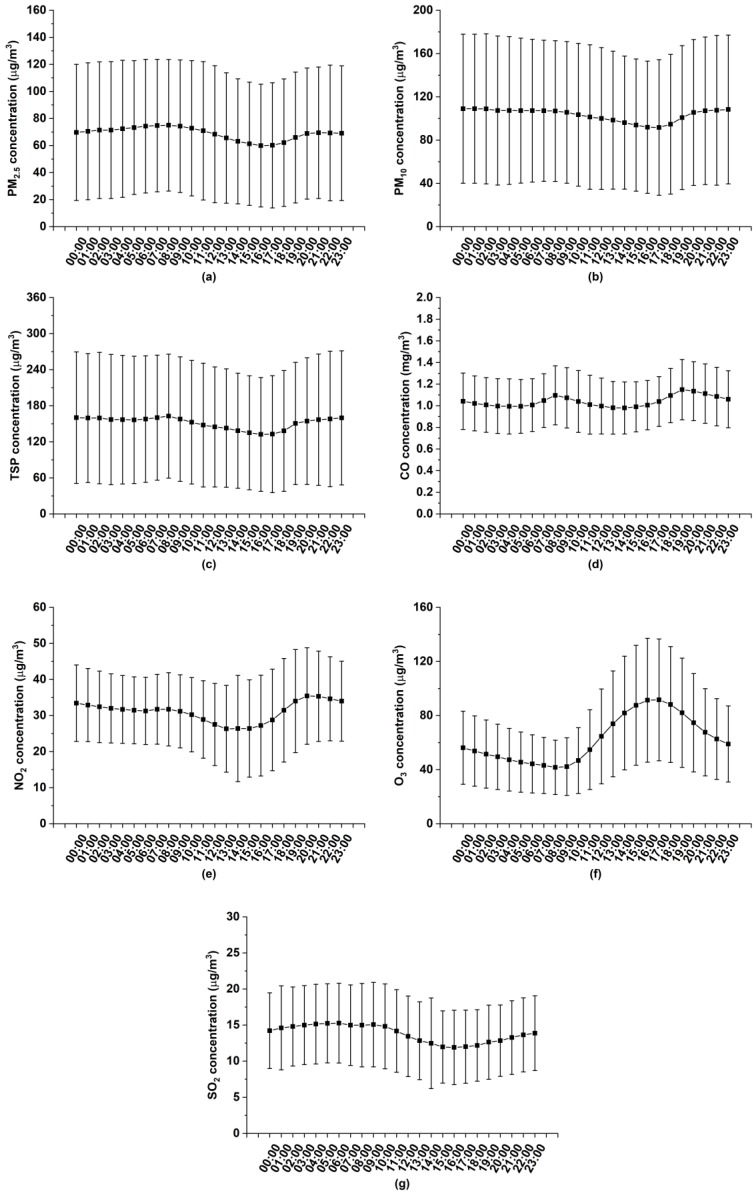

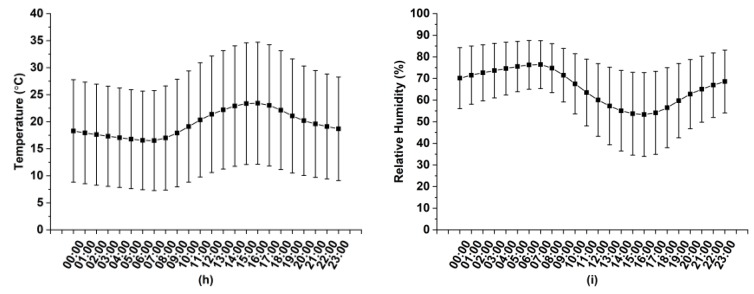
Daily variations of mean values of air pollutants, temperature and relative humidity in Xiangyang from March 1, 2018 to February 28, 2019: (**a**) PM_2.5_; (**b**) PM_10_; (**c**) TSP; (**d**) CO; (**e**) NO_2_; (**f**) O_3_; (**g**) SO_2_; (**h**) temperature; (**i**) relative humidity (The bars indicate mean value ± standard deviation).

Diurnal variations of air pollutants are shown in Figure 7. The diurnal characteristics of CO remain at a relatively steady status, despite two slight growths linked to the morning and evening rush hours. PM2.5, PM10, TSP, NO2 and SO2 enjoy similar diurnal characteristics with traffic-related double uptrends (5:00–8:00 and 17:00–20:00), and one downtrend (9:00–17:00), mainly ascribed to the change of PBLH. According to prior studies, the normal distributed PBLH increases from early in the morning (around 5:00 a.m.) to the noon, and then decreases to the minimum at night [14,19]. That is to say, the alleviated pollutant burden from noon to the afternoon results in the growth of PBLH by providing more volume to disperse pollutants. However, the decreases are not as drastic as expected. One possible reason for that is the counteractive effect of a synchronous decline of RH (Figure 7), which has adverse impacts on getting particles together to deposit. From the evening to the next day sunrise, lower PBLH enhances the concentrations of those pollutants, spontaneously leading to a high level of overnight pollution concentration, despite less anthropogenic emissions during that period. Interestingly, the diurnal variation of O3 concentration exhibits a thoroughly reverse pattern with a dramatic increase from 9:00 to 17:00, followed by a pronounced decrease persisting until the next day morning. Higher temperature at noon and in the afternoon (Figure 7) provides favorable conditions for photochemical activities accompanied by consumptions of NO2 and CO to generate O3. Furthermore, tails (20:00–23:00) of particulate trends vary from each other: (1) PM2.5 concentration gradually drops; (2) PM10 concentration maintains a stable condition; (3) TSP continues its growth till the next day morning. The on-road diesel trucks are permitted to transport construction waste from 22:00 to midnight according to relevant governmental regulations [36] and they contribute to the abundance of coarse particles accompanied by six-fold emissions more than those of other vehicles [14,45].

### 3.4. Relationships between Air Pollutants and Meteorological Parameters

**Figure 8:**
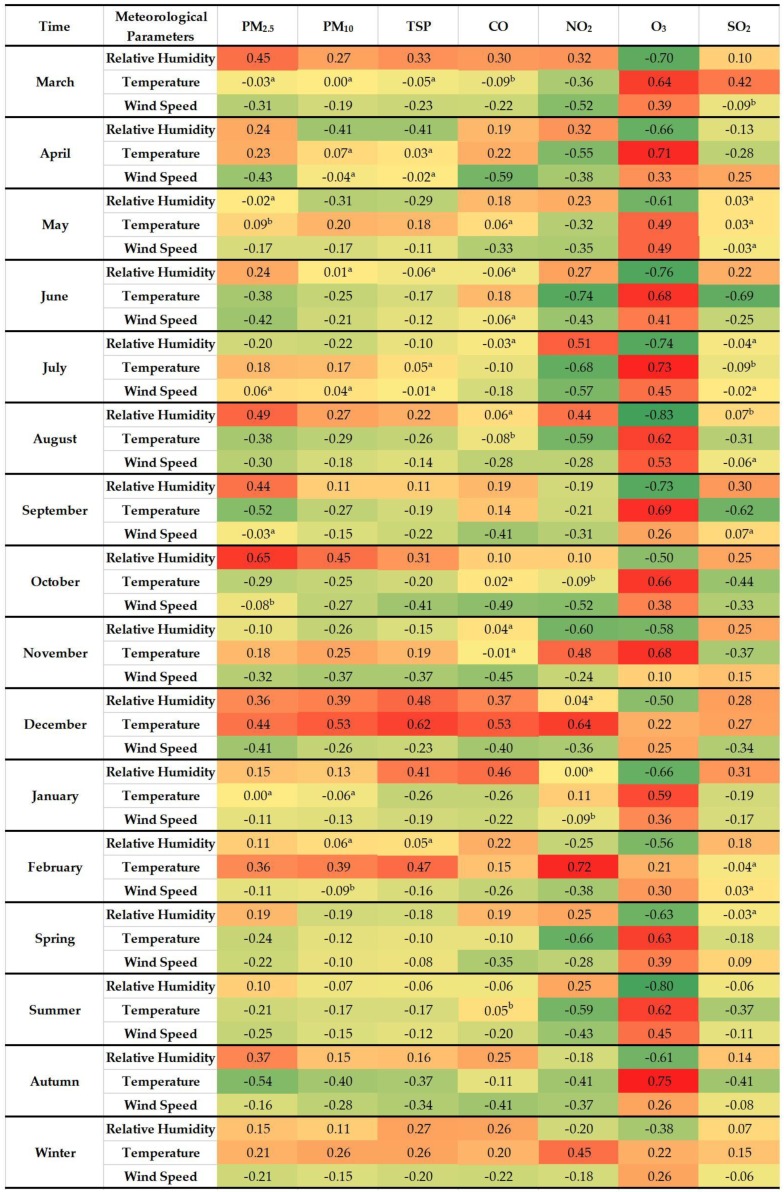
Spearman’s correlation coefficients between air pollutants and meteorological parameters. (All correlations are significant at 99% confidence interval. ^a^ Correlations are not significant. ^b^ Correlations are significant at 95% confidence interval. The darker the red, the stronger positive correlation and the darker the green, the stronger negative correlation.)

Figure 8 illustrates the relationships between seven air pollutants and three meteorological parameters. Generally, all correlation coefficients here are relatively lower than reported in previous studies, which could be caused by three possible reasons. The first reason can be different time resolutions between the data of air pollutants (hourly) and meteorological parameters (daily) in previous studies. The second reason is that air pollutants and meteorological parameters for the same city are measured by different stations situated at different locations. The last reason can be the nonlinear relationship between the studied air pollutants and meteorological parameters. RH positively correlates with PM_2.5_ in all seasons while showing evident season-changed (i.e., negative in spring and summer, positive in winter and autumn) correlations with coarse particles (i.e., PM_10_ and TSP). In warm seasons (i.e., spring and summer), a relatively larger amount of precipitation is reported, suggesting higher RH, and then the degree of wet deposition for hygroscopic particles is aggravated under such damp weathers, inducing the reduction of PM_10_ and TSP. Given the fact that RH positively correlates with PM_2.5_ in all seasons, we assert that RH does not play an evident role in scavenging fine particles. Nevertheless, a different mechanism is set up in cold seasons. Normally, RH is closely associated with aerosols in winter and autumn, helping aerosol precursors convert to secondary aerosol, which in turn magnifies the concentration of PM_2.5_ [19,46,47]. This also explains the relatively higher correlation coefficient between RH and PM_2.5_ than those between RH and coarse particles. As for the correlation coefficients of temperature with particulate pollutants, negative correlation coefficients appear from June to October (without July) and positive correlation coefficients display since November until February of the next year. The negative correlation correlations are due to better dispersion as well as wet deposition by the joint effect of more precipitations, stronger wind, thicker PBLH and better vertical air mixing.

In cold seasons, on the one hand, particles can take advantage of meteorological characteristics to accumulate, and on the other, enhanced secondary particulate pollutants, involving SO_2_ consumption, are generated and further sustained by the positive correlation between temperature and SO_2_ in some months. Moreover, higher temperature is beneficial to photochemical reactions. Therefore, temperature positively correlates with O_3_, especially in warm seasons, while negatively correlates with CO. Lower temperature and less sunlight inevitably hamper the evaporation effect, and therefore bring higher RH.

**Figure 9:**
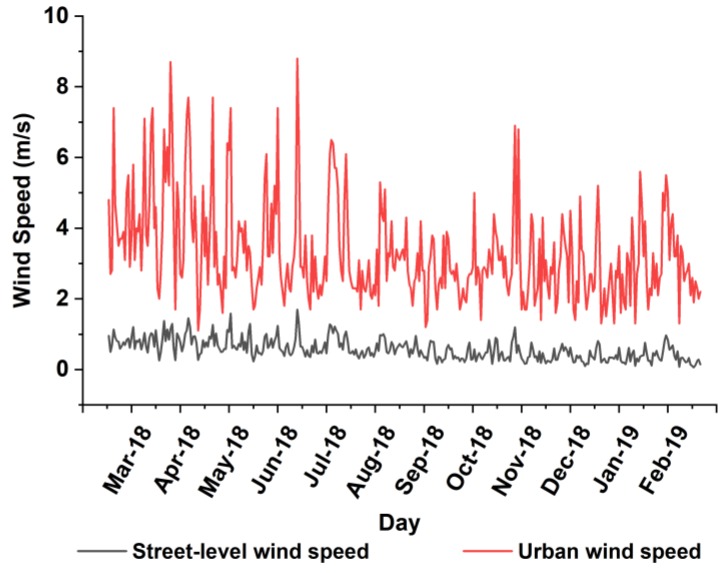
Comparisons of monthly mean variations for street-level wind speed (collected by 16 measurement station) and urban wind speed (collected by the China Meteorology Administration) from 1 March 2018 to 28 February 2019.

This probably explains the relatively stronger positive correlation coefficients of RH with CO and with SO_2_ during those seasons. The variability of the correlation between temperature and NO_2_ lies in the complex mechanism between NO_2_ and O_3_ in different meteorological characteristics. That is to say, the formation of secondary O_3_ by processing NO_2_ is relatively stronger in spring and summer while the oxidation from NO_x_ to NO_2_ is relatively stronger in autumn and winter. In terms of WS, all air pollutants, excluding O_3_, display inverse correlations in most months, due to the higher WS the better dispersion of pollutants. Furthermore, among all correlations between WS and particulate pollutants, the correlations with coarse particles are relatively weaker, probably due to dust resuspended by the wind. Yet, the strong positive correlation with O_3_ may result in regional transported O_3_ [48]. Notably, the correlation coefficients of WS with all air pollutants here are relatively lower than those of previous studies [18,19,23]. Apart from a possibility of the nonlinear relationship, another possible reason is different WS measurements between our study and previous studies. Specifically, the WS applied in our study is of street-level wind which is remarkably slowed down by high-density infrastructures in the built environment while the WS used in previous studies is of urban wind, despite similarities in fluctuations (Figure 9).

### 3.5. Implications and Limitations

Like Xiangyang, many prefecture-level cities whose GDP mainly relies on industrial production are confronting potent hazy weather as much as key cities. However, limited attention has been paid to this phenomenon. Therefore, our study seeks to provide a holistic and systematical examination of the spatiotemporal variations of both particulate and gaseous pollutants as well as on their relations to meteorological parameters for prefecture-level cities, which can be fully used by policy-makers to take accurate measures to mitigate the haze problem. Meanwhile, we reasonably demonstrated that it is imperative that air pollutants and meteorological parameters be synchronously monitored through ground measurements. Furthermore, this all-sided study can fill in the gap in the field of spatiotemporal variations of air pollutants at an urban scale. Nevertheless, our study has some limitations. First, one-year measurements are insufficient for analyses of seasonal variations and holiday effect, as longer time span measurements can rule out some uncertainties. Second, some meteorological factors, such as precipitations, sunshine durations and the PBLH, have not been participated in the Spearman’s rank correlation coefficient test in our study as unable to be synchronously measured by those 16 stations. Nevertheless, they can directly and indirectly disrupt the pollution concentration that was demonstrated by other studies [21,49,50,51,52]. Therefore, more monitoring parameters of ground stations and a precise measurement of PBLH for Xiangyang are well needed. Finally, further investigations on whether the relationship between the studied air pollutants and meteorological parameters is nonlinear or not are requested.

## 4. Conclusions

In this paper, we analyzed the spatiotemporal variations of particulate and gaseous pollutants and their relations to meteorological parameters in Xiangyang, China, from 1 March 2018 to 28 February 2019. The principal conclusions are as follows:

(1) The percentage of the days with excellent and good air quality (64.6%) fails to reach the criteria of 80% proposed by China’s 13th Five-Year Plan (2016–2020). PM_2.5_, O_3_ and PM_10_ are the main primary pollutants. Moreover, PM_2.5_ and PM_10_ act as primary pollutants all the year round and O_3_ loses its primary pollutant position after November.
(2) Annual concentrations of PM_2.5_ and PM_10_ of all stations strikingly exceed both the CNAAQS and WHOAQG. The largest abundance of gaseous pollutants is identically from Station 15, surrounded by nine chemical plants while the maximal enhancement of particulate pollutants is observed at Station 6, ascribed to its land use for automobile manufacture. TSP concentration is closely related to on-going construction sites but can be alleviated by road sprinkler operations.
(3) A distinct seasonal trend for “worse winter and better summer” is unveiled. However, the concentration of CO maintains a relatively stable condition in the whole year. Concentrations of PM_2.5_, PM_10_, TSP, NO_2_ and SO_2_ enjoy similar two traffic-related uptrends (5:00–8:00 and 17:00–20:00) and one downtrend (9:00–17:00), while the concentration of O_3_ fluctuates oppositely due to photochemical reactions.
(4) An unclear weekend effect is confirmed for all pollutants. Significant differences of NO_2_ and SO_2_ during NDH and non-NDH as well as significant differences of NO_2_ and O_3_ during CNY and non-CNY are unveiled. Celebration activities for CNY bring distinctly increased concentrations of SO_2_ and secondary particulate pollutants.
(5) RH positively correlates with PM_2.5_ in all seasons while showing evident season-changed (i.e., negative in spring and summer, positive in winter and autumn) correlations with coarse particles. Positive correlation coefficients of temperature with O_3_ appear in all seasons, and positive correlation coefficients of temperature with all other pollutants are shown only in winter. WS positively correlates with O_3_ while negatively correlates with the rest.

In the future, quantitative efforts to clarify the air pollution distribution in the built environment, and simultaneously to take into consideration with more urban features such as building density, percentage of greenery coverage, etc. need to be made.

## Figures and Tables

**Table 1:** Measurement site description and statistical summaries of hourly measurements for meteorological parameters in Xiangyang from 1 March 2018 to 28 February 2019.

Station	Measurement Site Description	Temperature (°C)		Relative Humidity (%)		Wind Speed (m/s)		Prevailing Wind Direction
		Min~Max	Hourly Average ± S.D.	Min~Max	Hourly Average ± S.D.	Min~Max	Hourly Average ± S.D.	
1	Residential and administrative	−2~43	19 ± 10	11~99	68 ± 19	0~9.0	1.4 ± 1.2	East-Southeast
2	Residential and close to a reservoir	−2~42	20 ± 11	11~99	66 ± 19	0~2.7	0.5 ± 0.4	Southwest
3	Close to a reservoir and a construction site	−2~47	20 ± 11	9~99	65 ± 18	0~3.8	0.4 ± 0.6	South-Southwest
4	Industrial	−2~47	20 ± 11	12~99	65 ± 18	0~1.9	0.3 ± 0.3	East-Southeast
5	Residential and surrounded by industrial factories	−2~42	19 ± 11	11~99	65 ± 18	0~2.4	0.4 ± 0.4	North-Northwest
6	Industrial	−2~46	21 ± 11	8~99	62 ± 19	0~3.1	0.8 ± 0.4	Northwest
7	Residential	−2~41	20 ± 10	13~99	66 ± 18	0~2.5	0.3 ± 0.4	Northwest
8	Residential of an urban center and close to Station 9, Station 12 and Station 13	−1~41	20 ± 11	11~99	64 ± 17	0~1.8	0.3 ± 0.3	Northeast
9	Residential of an urban center and close to Station 8, Station 12 and Station 13	−1~45	20 ± 11	10~99	63 ± 18	0~2.5	0.6 ± 0.6	South-Southeast
10	Administrative and residential of an urban center	−3~46	19 ± 11	10~99	68 ± 20	0~5.6	0.9 ± 0.8	North-Northeast
11	Administrative and residential of an urban center	−2~43	19 ± 10	12~99	69 ± 17	0~3.5	0.6 ± 0.6	South-Southwest
12	Residential of an urban center and close to Station 8, Station 9 and Station 13	−2~43	20 ± 11	10~98	65 ± 18	0~2.8	0.4 ± 0.4	Southwest
13	Residential of an urban center and close to Station 8, Station 9 and Station 12	−2~41	20 ± 11	12~99	66 ± 18	0~2.8	0.5 ± 0.5	East
14	Residential and close to a road intersection	−2~42	20 ± 11	11~99	66 ± 18	0~5.8	0.8 ± 0.7	West-Southwest
15	Industrial and far away from Station 1–14	−3~38	19 ± 9	12~95	68 ± 18	/	/	/
16	Industrial and far away from Station 1–14	−3~41	19 ± 10	13~95	70 ± 17	/	/	/

**Table 2:** Statistical summaries of hourly measurements for air pollutants in Xiangyang from 1 March 2018 to 28 February 2019.

Station	PM_2.5_ (ug/m^3^)		PM_10_ (ug/m^3^)		Total Suspended Particles (TSP) (ug/m^3^)		CO (mg/m^3^)		NO_2_ (ug/m^3^)		O_3_ (ug/m^3^)		SO_2_ (ug/m^3^)	
	Min~Max	Hourly Average ± S.D.	Min~Max	Hourly Average ± S.D.	Min~Max	Hourly Average ± S.D.	Min~Max	Hourly Average ± S.D.	Min~Max	Hourly Average ± S.D.	Min~Max	Hourly Average ± S.D.	Min~Max	Hourly Average ± S.D.
1	4~388	71 ± 55	9~542	101 ± 70	14~954	143 ± 106	0.3~3.1	1.0 ± 0.3	3~112	32 ± 14	4~245	63 ± 39	2~62	11 ± 6
2	3~232	52 ± 29	8~555	82 ± 51	12~974	120 ± 88	0.4~2.8	1.0 ± 0.2	3~92	19 ± 10	2~246	60 ± 42	1~57	10 ± 6
3	3~398	77 ± 58	7~626	120 ± 88	12~1282	179 ± 135	0.3~3.2	1.0 ± 0.3	3~110	32 ± 15	3~201	61 ± 32	2~461	14 ± 11
4	4~391	74 ± 55	8~544	104 ± 66	15~956	145 ± 92	0.4~2	1.0 ± 0.1	2~48	19 ± 7	3~302	62 ± 43	0~101	12 ± 10
5	3~364	71 ± 52	8~549	101 ± 67	14~964	135 ± 96	0.4~4.3	1.0 ± 0.3	3~95	33 ± 14	3~215	61 ± 33	2~117	16 ± 10
6	5~446	79 ± 61	8~626	124 ± 91	12~1034	182 ± 132	0.4~2.4	1.0 ± 0.2	2~123	31 ± 15	4~520	62 ± 37	2~84	16 ± 9
7	3~323	65 ± 44	8~548	94 ± 59	12~961	140 ± 101	0.4~3.1	1.0 ± 0.3	2~122	31 ± 16	2~214	62 ± 38	2~69	14 ± 7
8	4~350	69 ± 48	7~555	102 ± 67	13~976	147 ± 106	0.4~3.4	1.4 ± 0.5	2~109	33 ± 14	2~260	62 ± 39	2~86	13 ± 7
9	4~362	68 ± 51	7~549	102 ± 72	13~968	150 ± 111	0.6~4.6	1.0 ± 0.3	2~110	34 ± 14	3~244	62 ± 39	2~70	14 ± 8
10	3~330	65 ± 46	7~557	100 ± 69	11~2812	139 ± 109	0.2~3.1	1.0 ± 0.3	2~104	32 ± 14	2~399	63 ± 40	1~56	11 ± 6
11	4~375	72 ± 52	8~570	104 ± 70	16~992	158 ± 116	0.4~3.1	1.0 ± 0.3	2~148	32 ± 15	3~267	63 ± 39	2~64	12 ± 6
12	4~327	67 ± 45	8~551	97 ± 61	13~966	153 ± 111	0.1~3.8	0.8 ± 0.3	2~104	34 ± 13	3~241	62 ± 40	2~67	13 ± 6
13	0~399	73 ± 55	8~569	105 ± 73	14~998	156 ± 114	0.1~4	1.0 ± 0.3	3~107	35 ± 14	2~253	58 ± 41	2~76	15 ± 9
14	3~376	69 ± 52	8~570	105 ± 72	13~1001	172 ± 121	0.3~6.9	1.0 ± 0.3	2~89	32 ± 14	3~229	63 ± 39	2~65	11 ± 6
15	6~403	64 ± 53	4~508	101 ± 65	/	/	0.3~14.5	1.4 ± 1.8	1~176	35 ± 24	1~295	72 ± 52	1~396	23 ± 50
16	3~637	67 ± 53	17~848	110 ± 71	/	/	0.4~7.5	1.0 ± 0.4	1~147	33 ± 19	1~291	65 ± 42	0~675	15 ± 15

**Table 3:** The air quality index (AQI) and corresponding health effects.

AQI	Levels	Air Quality Conditions	Health Effects
0–50	I	Excellent	People are free from air pollution.
51–100	II	Good	Some pollutants may have a weak health impact on a tiny minority of people with unusual sensitivity.
101–150	III	Slightly Polluted	Symptoms in susceptible crowd are slightly aggravated; symptoms of irritation appear in healthy crowd.
151–200	IV	Moderately Polluted	Symptoms in susceptible crowd are further aggravated; it may have an impact on the cardiac system and/or respiratory system of healthy crowds.
201–300	V	Heavily Polluted	Patients with heart or lung diseases have significantly increased symptoms and will experience reduced exercise tolerance; healthy crowd commonly experience health effect.
>300	VI	Severely Polluted	Healthy crowd will apparently have intense symptoms and experience reduced exercise tolerance; some diseases appear in advance.

**Table 4:** Annual mean concentrations of 16 measurement stations in Xiangyang from 1 March 2018 to 28 February 2019.

Station	Annual Mean Concentrations		Station	Annual Mean Concentrations		Station	Annual Mean Concentrations		Station	Annual Mean Concentrations	
1	PM_2.5_	71.1	2	PM_2.5_	*52.0*	3	PM_2.5_	76.5	4	PM_2.5_	74.4
	PM_10_	100.9		PM_10_	*81.7*		PM_10_	119.8		PM_10_	104.3
	TSP	143.2		TSP	*119.9*		TSP	178.9		TSP	144.6
	CO	1.0		CO	1.0		CO	1.0		CO	1.0
	NO_2_	32.2		NO_2_	18.8		NO_2_	31.7		NO_2_	*18.8*
	O_3_	63.0		O_3_	59.9		O_3_	61.1		O_3_	62.3
	SO_2_	11.3		SO_2_	*9.8*		SO_2_	14.2		SO_2_	11.6
5	PM_2.5_	71.0	6	PM_2.5_	**79.1**	7	PM_2.5_	64.8	8	PM_2.5_	69.0
	PM_10_	100.7		PM_10_	**124.1**		PM_10_	94.0		PM_10_	101.5
	TSP	135.0		TSP	**181.7**		TSP	139.9		TSP	147.3
	CO	1.0		CO	1.0		CO	1.0		CO	1.4
	NO_2_	33.3		NO_2_	31.5		NO_2_	31.5		NO_2_	33.4
	O_3_	61.3		O_3_	62.0		O_3_	62.3		O_3_	62.3
	SO_2_	15.9		SO_2_	16.4		SO_2_	14.2		SO_2_	13.3
9	PM_2.5_	67.8	10	PM_2.5_	65.2	11	PM_2.5_	71.6	12	PM_2.5_	66.9
	PM_10_	102.1		PM_10_	100.5		PM_10_	103.7		PM_10_	97.4
	TSP	150.2		TSP	139.5		TSP	157.7		TSP	153.1
	CO	1.0		CO	1.0		CO	1.0		CO	*0.8*
	NO_2_	33.7		NO_2_	31.9		NO_2_	32.4		NO_2_	33.7
	O_3_	61.8		O_3_	63.3		O_3_	62.9		O_3_	62.2
	SO_2_	14.4		SO_2_	11.0		SO_2_	12.0		SO_2_	13.5
13	PM_2.5_	72.5	14	PM_2.5_	69.0	15	PM_2.5_	64.4	16	PM_2.5_	66.7
	PM_10_	105.0		PM_10_	104.6		PM_10_	101.1		PM_10_	110.0
	TSP	156.1		TSP	171.7		TSP	**/**		TSP	**/**
	CO	1.0		CO	1.0		CO	**1.4**		CO	1.0
	NO_2_	34.7		NO_2_	31.9		NO_2_	**35.6**		NO_2_	32.8
	O_3_	*58.2*		O_3_	62.6		O_3_	**72.0**		O_3_	64.7
	SO_2_	14.8		SO_2_	11.2		SO_2_	**23.4**		SO_2_	14.6

The unit of CO is mg/m^3^. Units of PM_2.5_, PM_10_, TSP, NO_2_, O_3_ and SO_2_ are ug/m^3^. Underlined italic numbers are the minimal values. Underlined bold numbers are the maximal values.

**Table 5:** Results of holiday-nonholiday difference tests at 16 measurement stations.

Difference Test	Air Pollutant	*p* Value	^2^ *H*_0_	^3^ Mean Concentrations during Non-Holiday Period	^3^ Mean Concentrations during Holidays
^1^ NDH-nonNDH	PM_2.5_	0.29	Accept	109.57	43.39
	PM_10_	0.73	Accept	147.23	74.71
	TSP	0.74	Accept	194.9	109.98
	CO	0.36	Accept	1.13	0.93
	NO_2_	0.03	***Reject***	28.9	42.98
	O_3_	0.13	Accept	43.41	63.62
	SO_2_	0.04	***Reject***	12.74	15.13
^1^ CNY-nonCNY	PM_2.5_	0.48	Accept	122.46	109.57
	PM_10_	0.57	Accept	164.35	147.23
	TSP	0.30	Accept	235.55	194.9
	CO	0.20	Accept	1.22	1.13
	NO_2_	0	***Reject***	40.93	28.9
	O_3_	0	***Reject***	28.06	43.41
	SO_2_	0.17	Accept	13.87	12.74

^1^ NDH denotes National Day holidays and CNY denotes Chinese New Year. ^2^ The confidence interval is 95%. ^3^ The unit of CO is mg/m^3^. Units of PM_2.5_, PM_10_, TSP, NO_2_, O_3_ and SO_2_ are ug/m^3^.

**Table 6:** Results of weekday-weekend statistical difference tests at 16 measurement stations.

Air Pollutant	*p* Value	*H*_0_^1^	Mean Concentration on Weekdays ^2^	Mean Concentration on Weekends ^2^
PM_2.5_	0.70	Accept	71.67	73.68
PM_10_	0.05	Accept	108.64	104.70
TSP	0.44	Accept	159.01	156.31
CO	0.12	Accept	1.05	1.08
NO_2_	0.70	Accept	31.42	31.15
O_3_	0.25	Accept	63.55	62.15
SO_2_	0.05	Accept	14.14	13.47

^1 ^The confidence interval is 95%. ^2 ^The unit of CO is mg/m^3 ^and units of PM_2.5_, PM_10_, TSP, NO_2_, O_3_ and SO_2_ are ug/m^3^.
